# ACL reconstruction combined with lateral monoloop tenodesis can restore intact knee laxity

**DOI:** 10.1007/s00167-019-05839-y

**Published:** 2020-01-25

**Authors:** Koen C. Lagae, Joris Robberecht, Kiron K. Athwal, Peter C. M. Verdonk, Andrew A. Amis

**Affiliations:** 1 Antwerp Orthopaedic Centre Monica Hospitals, Antwerp, Belgium; 2 Galeazzi Orthopaedic Institute, Milan, Italy; 3 Department of Orthopaedic Surgery, Antwerp University Hospital (UZA), Antwerp, Belgium; 4 Biomechanics Group, Department of Mechanical Engineering, Imperial College London, London, SW7 2AZ UK; 5 Musculoskeletal Surgery Group, Imperial College London School of Medicine, Charing Cross Hospital, London, W6 8RF UK

**Keywords:** ACL reconstruction, Monoloop, Knee laxity, Biomechanics, Lateral extra-articular tenodesis, Anterolateral rotational instability

## Abstract

**Purpose:**

An anterior cruciate ligament (ACL) injury is often combined with injury to the lateral extra-articular structures, which may cause a combined anterior and rotational laxity. It was hypothesised that addition of a ‘monoloop’ lateral extra-articular tenodesis (mLET) to an ACL reconstruction would restore anteroposterior, internal rotation and pivot-shift laxities better than isolated ACL reconstruction in combined injuries.

**Method:**

Twelve cadaveric knees were tested, using an optical tracking system to record the kinematics through 0°–100° of knee flexion with no load, anterior and posterior translational forces (90 N), internal and external rotational torques (5 Nm), and a combination of an anterior translational (90 N) plus internal rotational load (5 Nm). They were tested intact, after sectioning the ACL, sectioning anterolateral ligament (ALL), iliotibial band (ITB) graft harvest, releasing deep ITB fibres, hamstrings tendon ACL reconstruction, mLET combined with ACL reconstruction, and isolated mLET. Two-way repeated-measures ANOVA compared laxity data across knee states and flexion angles. When differences were found, paired *t* tests with Bonferroni correction were performed.

**Results:**

In the ACL-deficient knee, cutting the ALL significantly increased anterior laxity only at 20°–30°, and only significantly increased internal rotation at 50°. Additional deep ITB release significantly increased anterior laxity at 40°–90° and caused a large increase of internal rotation at 20°–100°. Isolated ACL reconstruction restored anterior drawer, but significant differences remained in internal rotation at 30°–100°. After adding an mLET there were no remaining differences with anterior translation or internal rotation compared to the intact knee. With the combined injury, isolated mLET allowed abnormal anterior translation and rotation to persist.

**Conclusions:**

Cutting the deep fibres of the ITB caused large increases in tibial internal rotation laxity across the range of knee flexion, while cutting the ALL alone did not. With ACL deficiency combined with anterolateral deficiency, ACL reconstruction alone was insufficient to restore native knee rotational laxity. However, combining a ‘monoloop’ lateral extra-articular tenodesis with ACL reconstruction did restore native knee laxity.

## Introduction

Anterior cruciate ligament (ACL) injury causes a ‘pivot-shift’ instability, which is a combination of increased anterior translation and internal rotation of the tibia [4, 26]. Among ACL-injured knees there is a range of the amount of rotational instability, which is believed to be caused by additional injury to the anterolateral soft tissue structures [27, 37, 38]. This view is supported by radiological findings of the Segond fracture and concomitant injury to the anterolateral soft tissues at the time of injury [14, 24, 41].

The main anterolateral structures controlling rotational stability are the superficial and deep fibres of the iliotibial band (ITB) and the anterolateral ligament (ALL) complex [18, 21, 37]. In recent literature, focus has been on the anterolateral ligament for controlling rotational laxity [27]. However, it is not well understood how this compares to the superficial and deep fibres of the ITB, which are important structures regarding the control of rotational laxity [21, 39, 42]. A recent robotic sectioning study found that sectioning the Kaplan fibres had a greater impact on rotational laxity when compared with ALL sectioning [11].

Historically, an isolated lateral extra-articular tenodesis (LET) alone was used to stabilise ACL-deficient knees. This isolated LET surgery, originally described as a procedure according to Lemaire or MacIntosh, used an 18 cm-long strip of ITB originating at Gerdy’s tubercle, tunneled deep to the LCL, fixed on the distal femur proximal and posterior to the lateral epicondyle (Lemaire) or around the distal Kaplan fibres (MacIntosh), returning deep to the LCL, with the end of the graft fixed on itself using sutures and sometimes secured to the tibia near Gerdy’s tubercle [1, 23]. Unfortunately, follow-up results showed inferior outcomes and it was suggested that, due to overconstraining the lateral compartment, lateral osteoarthritis could occur [9, 28, 33].

Currently, ACL injuries are mostly treated with an intra-articular ACL reconstruction.

Although this procedure provides acceptable restoration of tibial anterior translation laxity, in some cases residual rotational laxity or instability can persist [4, 10, 31]. In case of an isolated ACL deficiency with intact anterolateral structures, an ACL reconstruction alone may restore normal knee kinematics and additional extra-articular tenodesis is not beneficial [3]. However, an ACL reconstruction alone may not be able to restore normal kinematics following a combined ACL and anterolateral injury [17, 35]. These findings in combination with recent studies on the anterolateral complex, including the anterolateral ligament (ALL) [5], increased the interest in combining an intra-articular ACL reconstruction with an anterolateral procedure such as an LET or ALL reconstruction [8, 25, 29, 33, 34, 40, 43]. The advantage of combining an ACL reconstruction with an ALL reconstruction was doubted by Noyes and Barber [30], who only found a minor reduction in rotational laxity which would not warrant a routine ALL reconstruction in ACL + ALL deficient knees.

This study aimed to determine: (1) the effect of sequentially cutting the anterolateral structures on the translational and rotational laxity of the knee; (2) whether either an ACL reconstruction alone or a combined ACL reconstruction with a specific ‘monoloop’ LET (mLET) can restore native knee laxity in the case of a combined ACL plus anterolateral injury; and (3): whether an isolated mLET can restore native knee laxity in the case of a combined ACL plus anterolateral injury.

It was hypothesised that sectioning the ALL would have a limited influence on internal rotational and pivot-shift laxity and that an additional release of the deep fibres of the ITB would significantly increase the internal rotational and pivot-shift laxity.

It was also hypothesised that combining an ACL reconstruction with an mLET would restore native knee laxities in a combined ACL and anterolateral injury model.

The clinical usefulness of this study is that, if the hypotheses were to be supported by the experimental data, it would reinforce understanding of the effects of damage to the anterolateral structures and then demonstrate the efficacy of a surgical method to restore native knee laxities in both translation and rotation.

## Material and methods

Twelve fresh-frozen non-paired cadaveric knees (4 male and 8 female) of median age 64 years (range 46–79) were used (all left knees), with consent of the Imperial College Healthcare Tissue Bank, licence 12275, approval R17013. All specimens were stored at − 20 °C. After thawing, inspection with manual examination and arthroscopy, none of the knees showed signs of ligament injury, previous surgery or significant osteoarthritis. The number of specimens was based on previous work on knee ligament reconstructions using the same methods, which gave post hoc powers in the range 0.88–0.97 for translations and rotations [17].

The femoral and tibial shafts were cut at 200 mm from the joint line, and the soft tissues at 150 mm. Skin and subcutaneous fat were resected. The distal end of the fibula was fixed to the tibia with a cortical screw. The distal tibia was fixed in a 60 mm-diameter cylindrical steel pot with a 500 mm extending rod using polymethyl methacrylate (PMMA). The medullary canal of the femur was reamed and the femur was cemented onto an intramedullary rod.

The knee was mounted on a 6 degrees-of-freedom test rig with the femur in a fixed position of 6° of valgus, and the tibia hanging down unconstrained [16]. The rig allowed passive flexion–extension from 0° to 100°. A mediolateral Steinmann pin was placed through the proximal tibia to apply 90 N anterior and posterior drawer forces via semi-circular hoops. A horizontal disc (Ø 200 mm) was connected around the tibial rod, and rotational torques could be applied via a pulley-and-string system connecting two weights on either side to the horizontal disc (5 Nm internal–external torque). The neutral position of the tibia in relation to the femur, at any angle of knee flexion, was defined by the position of the free-hanging tibia with no loading imposed on it, only its own weight. A clamping device around the tibial rod was used to mark the neutral rotation at the start of the experiment so that the original neutral rotation could be returned to during the experiment.

### Measurements

Reflective markers (Brainlab) were mounted with bicortical rods on the tibia and femur. Fiducial marker screws were used to mark the medial and lateral epicondyles and proximal end of the femur, and the most medial and lateral points of the tibial plateau and distal tibial end. These landmarks were digitised with a stylus probe to define the femoral and tibial coordinate systems, respectively [16]. A Polaris optical tracking system (Vega, Northern Digital Inc) was used to track the motion of the tibia and femur throughout the experiment. The test method had been developed for previous studies [7, 17, 21, 22]. A parallel position of the tibial and femoral rods in the sagittal plane was defined as 0° flexion. The full six degrees-of-freedom motions were described as tibial movements relative to the femur. The tracking system had a translational accuracy of ± 0.1 mm, defined by Khadem et al. [19].

### Surgical technique

Surgery was performed by one surgeon whilst the knee was mounted on the test rig. During the experiment, the knee was kept moist by regularly spraying it with water. First, the gracilis and semitendinosus tendons were harvested and a 4.5 × 40 mm tibial post screw was placed. Thereafter, the intact knee was tested. An arthroscopy was performed to rule out damage to the cruciate ligaments, menisci and significant osteoarthritis. The ACL was cut and the tibial and femoral tunnels were drilled through the middle of the anatomical attachments, to obtain an ‘anatomical ACL reconstruction’ [15]. The femoral tunnel was drilled through the anteromedial portal. The drill size for the femoral and tibial tunnels was chosen according to the diameter of the tendon graft; this was normally 8 mm for both femoral and tibial tunnels.

**Fig. 1:**
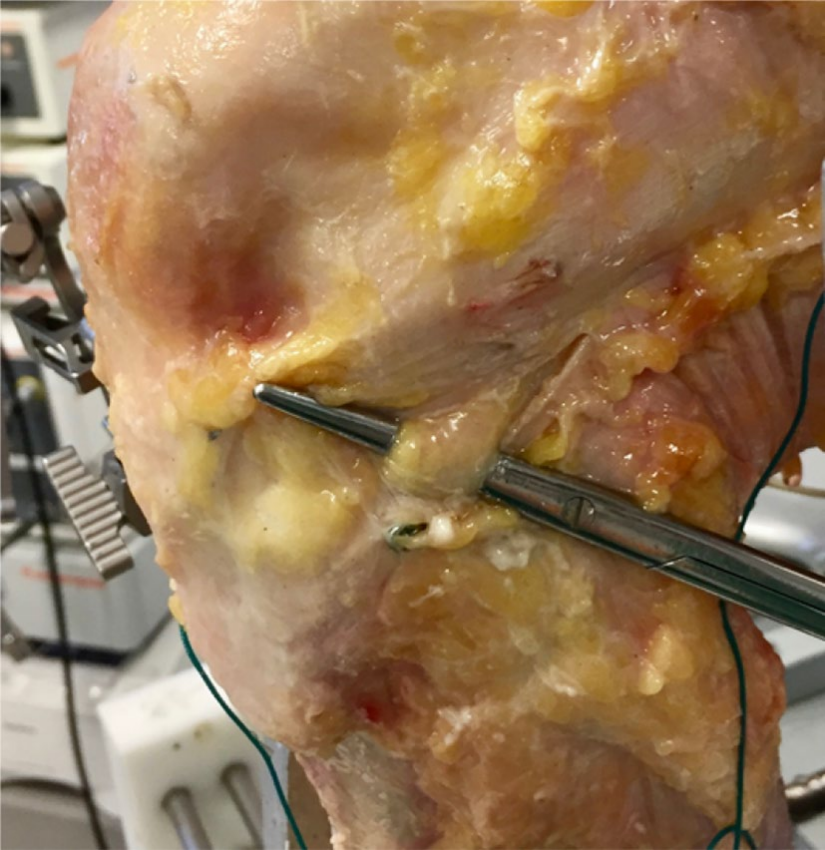
Isolating the ALL through a fibre splitting incision of the ITB

Once the ACL-deficient knee had been tested, the anterolateral structures were sectioned in the following order: first the ALL was palpated under the ITB. The ALL runs from close to the prominence of the lateral femoral epicondyle and passes over the LCL towards a point midway between Gerdy's tubercle and the tip of the fibular head [5, 18]. A 15-mm fibre splitting incision in the iliotibial band (ITB) was made overlying the anterolateral ligament (ALL) that gave easy access to the ALL and LCL without cutting any structures of the deep ITB, and the ALL was sectioned through this incision. Blunt dissection with the dissection scissors was performed between the ALL and the capsule (Fig. 1) and then the ALL was transected by cutting with a scalpel down onto the scissors. The deep ITB fibres are situated more proximally, so were undamaged. The knee was then retested.

**Fig. 2:**
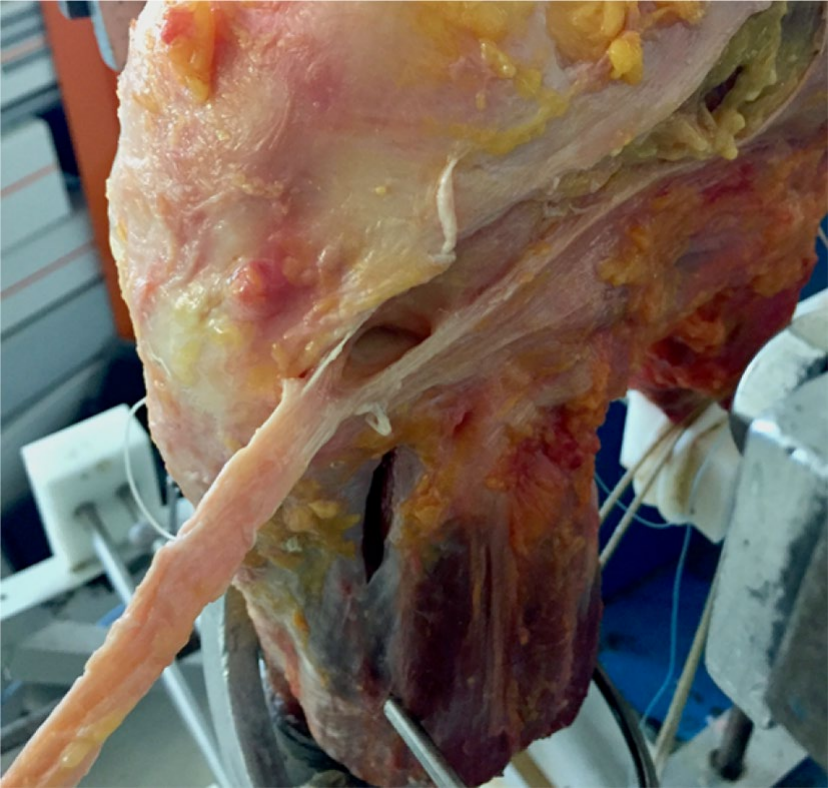
Harvesting the ITB strip

Next, a midportion ITB strip was harvested. The attachment to Gerdy’s tubercle was preserved and an ITB strip of 10 mm width × 150 mm length was taken in the middle of the anteroposterior width of the ITB (Fig. 2). The ITB strip was taken from the central part of the ITB, leaving the posterior part intact, which tightens when internally rotating the tibia, therefore adding to anterolateral stability. The knee was retested again.

Lastly, the deep fibres of the ITB were released. This was done by sectioning the connecting fibres between the ITB and the posterolateral surface of the distal femur. By doing this, the distal Kaplan fibres were also released [13, 21]. The knee laxity was measured after each stage.

Once these three anterolateral structures had been sectioned, the hamstrings tendon graft for the ACL (4-strand: semitendinosus + gracilis) was prepared and then inserted. The graft was fixed to the femur with a cortical suspensory fixation (Endobutton CL Ultra, Smith & Nephew). Fixation on the tibia used an 8 × 25 mm interference screw (BioRCI, Smith & Nephew) whilst a tension of 80 N was applied [16], with additional backup fixation by tying the whipstitched sutures (Ultrabraid, Smith & Nephew) to the tibial post screw. This ACL reconstructed knee was tested before adding a ‘monoloop’ lateral extra-articular tenodesis (mLET).

**Fig. 3:**
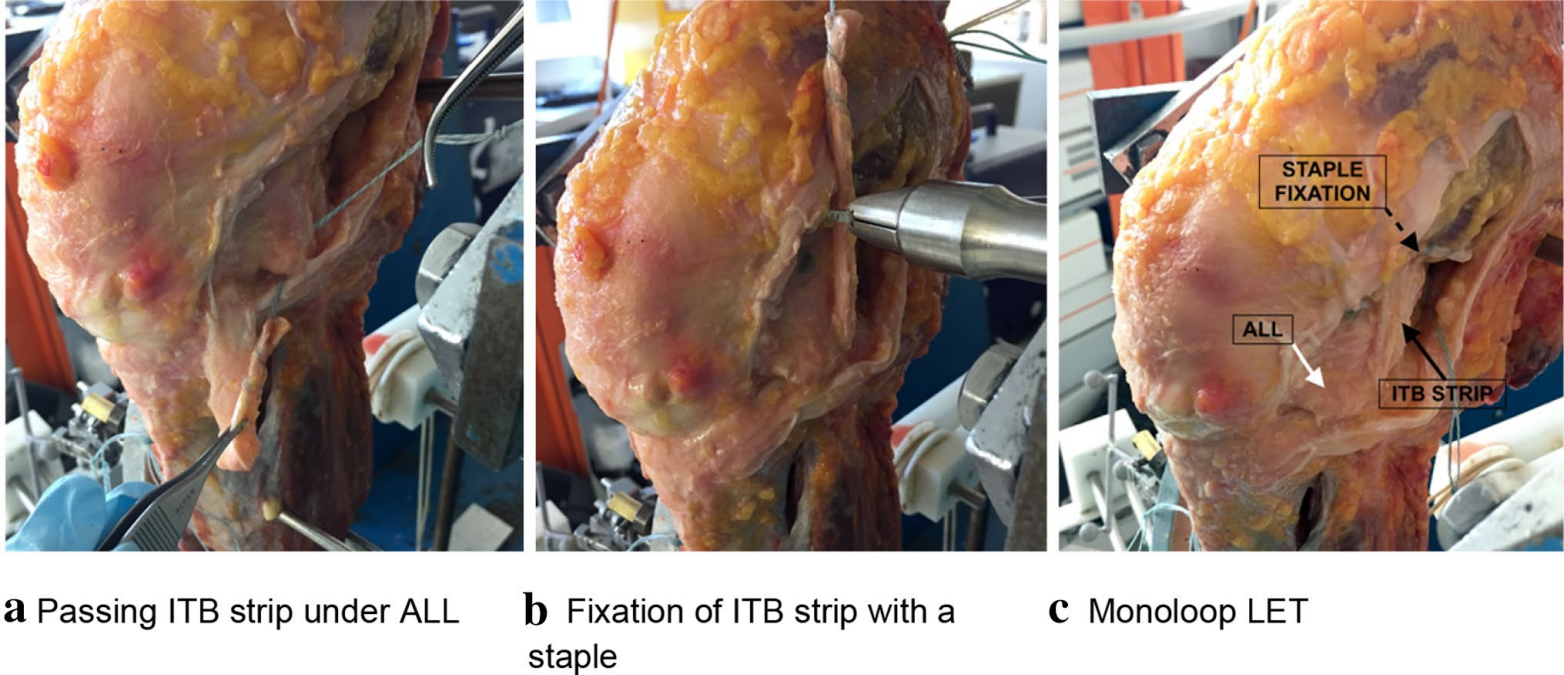
‘Monoloop’ lateral extra-articular tenodesis (mLET)

For the mLET procedure, the tibia was locked in the initial neutral rotation defined by the clamping device, and the single ITB strip (termed ‘monoloop’) 10 mm wide and 150 mm long was routed deep to the LCL and fixed with a 12 × 23 mm staple (Smith & Nephew: Richards Regular Fixation Staples with spikes, USA) on the distal and posterior aspect of the femoral shaft. The fixation site of the mLET was just proximal to the insertion of the intermuscular septum at the distal femur. The key to this technique is to bring the graft just posterior to the LCL, deep to the soft tissues and, even sometimes depending on anatomy, deep to fibres of the lateral gastrocnemius and deep to the septum, to the exact location of the deep fibres of the ITB. It would always be in the posterior half of the femur on lateral X-ray views and proximal to the endobutton fixation device on the lateral cortex of the femoral shaft. This tenodesis was fixed at an applied tension of 20 N at 60° knee flexion and neutral tibial rotation (Fig. 3).

The knee was tested with the combined ACL reconstruction and mLET in place and then, after the ACL graft was removed, the knee was tested with the isolated mLET in place. This testing order was not randomised because that would have demanded a greater number of knees to maintain statistical power for little important extra data.

### Testing protocol

Each state of the knee was tested by cycling it through 0° to 110° of passive flexion and recording the kinematic data. Three cycles in each of the following conditions were performed: without external forces (‘neutral’), 90 N anterior drawer force, 90 N posterior drawer force, 5 Nm external rotation torque, 5 Nm internal rotation torque, and combined 5 Nm internal rotation torque plus 90 N anterior drawer force.

### Statistical analysis

MATLAB scripts (The MathWorks Inc) were used for processing data and calculating tibial translations and rotations at 10° intervals throughout 0°–100° of knee flexion. For statistical processing, SPSS (version 24, IBM Corp.) was used. The Shapiro–Wilk test confirmed that the data sets were normally distributed. An a priori significance level of 0.05 was used to denote statistical significance. Two-way repeated-measures analyses of variance (ANOVAs) were used to compare the dependent laxity data (translational and rotational measures) across the two independent variables: state of the knee (intact, ACL deficient, ACL + ALL deficient, etc.) and flexion angle (0°, 10°, 20°, etc.). Paired *t* tests with Bonferroni correction were applied when differences across test conditions were found.

## Results

### The effect of ACL, anterolateral ligament and ITB sectioning on tibial anterior translation laxity

**Fig. 4:**
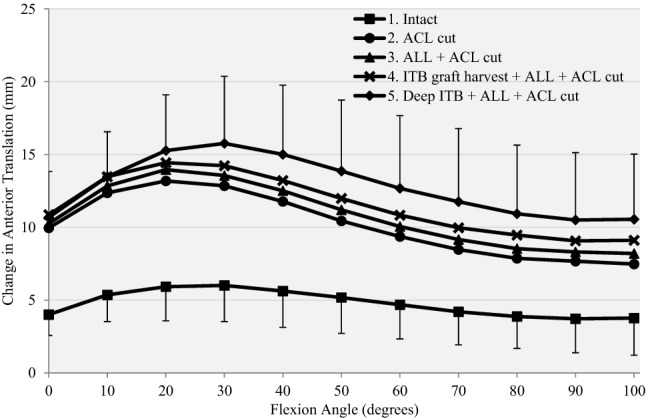
Response to a 90-N anterior drawer force for (1) intact, (2) anterior cruciate ligament deficient (ACL Cut), (3) ACL deficient and section of anterolateral ligament (ACL + ALL cut), (4) ACL deficient, section of ALL and ITB graft harvested (ITB graft harvest + ALL + ACL cut) and (5) release of deep ITB, section of ALL, ACL and ITB graft harvested (deep ITB + ALL + ACL cut)

Cutting the ACL significantly increased anterior translation laxity by a mean of 4–7 mm compared to the intact knee at all flexion angles (*p* < 0.01 from 0° to 90°, *p* = 0.013 at 100°). (Fig. 4, Table 1).

**Table 1:** Mean anterior translation ± standard deviation in mm, in response to 90 N anterior force (*n* = 12)

Flexion angle	Intact	ACL cut	ALL + ACL cut	ITB graft harvest + ALL + ACL cut	Deep ITB + ALL + ACL cut	ACL graft	mLET + ACL graft	mLET (no ACL graft)
0°	4.0 ± 1.4	10.0 ± 2.9^a^	10.3 ± 2.9^a^	10.9 ± 3.9^a^	10.7 ± 3.1^a^	5.2 ± 2.9	6.2 ± 2.8	10.2 ± 3.0^a^
10°	5.4 ± 1.8	12.4 ± 2.1^a^	12.8 ± 1.9^a^	13.5 ± 2.8^a^	13.5 ± 3.1^a^	7.1 ± 3.0	7.5 ± 2.3	12.3 ± 2.9^a^
20°	5.9 ± 2.3	13.2 ± 2.2^a^	14.0 ± 2.2^a,b^	14.4 ± 2.9^a^	15.3 ± 3.8^a,b^	8.1 ± 3.7	8.1 ± 2.3	13.6 ± 3.3^a^
30°	6.0 ± 2.5	12.9 ± 2.5^a^	13.6 ± 2.7^a,b^	14.2 ± 3.5^a^	15.8 ± 4.6^a,b^	8.6 ± 4.9	8.2 ± 2.5	13.7 ± 4.0^a^
40°	5.6 ± 2.5	11.8 ± 2.7^a^	12.5 ± 3.1^a^	13.2 ± 3.8^a^	15.0 ± 4.8^a,b,c^	8.5 ± 5.3	8.0 ± 2.6	13.0 ± 4.5^a^
50°	5.2 ± 2.5	10.4 ± 2.9^a^	11.2 ± 3.3^a^	12.0 ± 4.1^a^	13.9 ± 4.9^a,b,c,d^	7.9 ± 5.6	7.1 ± 2.7	11.9 ± 4.7^a^
60°	4.7 ± 2.4	9.4 ± 2.7^a^	10.1 ± 3.3^a^	10.8 ± 4.2^a^	12.7 ± 5.0^a,b,c,d^	7.4 ± 5.8	6.3 ± 2.4	10.8 ± 4.6^a^
70°	4.2 ± 2.3	8.5 ± 2.6^a^	9.2 ± 3.3^a^	10.0 ± 4.0^a^	11.8 ± 5.0^a,b,c,d^	6.8 ± 5.8	5.4 ± 2.3	9.8 ± 4.4^a^
80°	3.9 ± 2.2	7.9 ± 2.6^a^	8.5 ± 2.9^a^	9.5 ± 3.9^a^	10.9 ± 4.7^a,b,c^	6.4 ± 5.5	4.7 ± 2.0	9.0 ± 4.2^a^
90°	3.7 ± 2.3	7.7 ± 2.5^a^	8.3 ± 3.1^a^	9.1 ± 3.8^a^	10.5 ± 4.6^a,b,c^	6.2 ± 5.0	4.1 ± 1.8	8.5 ± 3.8^a^
100°	3.8 ± 2.5	7.5 ± 2.7^a^	8.2 ± 2.9^a^	9.1 ± 3.7^a^	10.6 ± 4.5^a,b,c^	5.9 ± 5.2	3.8 ± 2.1	8.3 ± 3.8^a^

^a^Significant difference from intact state (*p* < 0.05)

^b^Significant difference from ACL cut state (*p* < 0.05)

^c^Significant difference from ALL + ACL cut state (*p* < 0.05)

^d^Significant difference from ITB graft harvest + ALL + ACL cut state (*p* < 0.05)

Further sectioning of the ALL increased anterior translation laxity by < 1 mm, significant at 20°–30° knee flexion compared to the ACL-deficient knee (*p* < 0.05).

Harvesting the midportion ITB strip did not affect anterior translation laxity significantly.

Additional sectioning of the deep ITB significantly increased anterior translation laxity by up to 3 mm, significant at 40° to 100° flexion compared to the ACL and ALL-deficient knee (*p* < 0.05).

After the cutting stages, the knees with combined soft tissue damage were significantly more lax in tibial anterior translation than the intact knee, by 7–10 mm across 0°–100° flexion (*p* ≤ 0.004).

There was no change in tibial posterior translation after cutting the ACL and anterolateral structure.

### The effect of ACL, anterolateral ligament and ITB sectioning on tibial internal rotation laxity

**Fig. 5:**
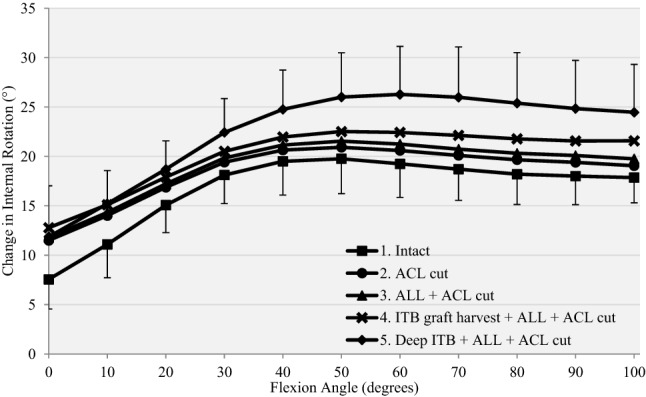
Response to a 5-Nm internal torque for (1) intact, (2) anterior cruciate ligament deficient (ACL Cut), (3) ACL deficient and section of ALL (ACL + ALL cut), (4) ACL deficient, section of ALL and ITB graft harvested (ITB graft harvest + ALL + ACL cut) and (5) release of deep ITB, section of ALL, ACL and ITB graft harvested (deep ITB + ALL + ACL cut)

Cutting the ACL did not increase tibial internal rotation laxity significantly compared to the intact knee at any flexion angle (mean changes 1°–4°). (Fig. 5, Table 2).

**Table 2:** Mean internal rotation ± standard deviation in degrees, in response to 5 Nm internal torque (*n* = 12)

Flexion angle	Intact	ACL cut	ALL + ACL cut	ITB graft harvest + ALL + ACL cut	Deep ITB + ALL + ACL cut	ACL graft	mLET + ACL graft	mLET (no ACL graft)
0°	7.5 ± 3.0	11.5 ± 4.2	11.8 ± 5.1	12.8 ± 5.3	11.9 ± 5.2	9.4 ± 5.2	10.1 ± 4.5	12.5 ± 6.0
10°	11.1 ± 3.4	14.0 ± 3.3	14.3 ± 3.8	15.1 ± 3.7	15.2 ± 3.4^a^	13.4 ± 3.1	12.5 ± 3.0	14.9 ± 3.7
20°	15.1 ± 2.8	16.9 ± 2.8	17.2 ± 3.0	17.9 ± 3.1^a^	18.7 ± 2.9^a,b,c,d^	17.8 ± 2.7	15.9 ± 3.1^e^	17.9 ± 3.2
30°	18.1 ± 2.9	19.4 ± 3.1	19.8 ± 3.4	20.5 ± 3.8^a^	22.4 ± 3.4^a,b,c,d^	21.4 ± 3.5^a^	18.8 ± 3.8^e^	21.2 ± 3.8^a^
40°	19.5 ± 3.4	20.6 ± 3.8	21.2 ± 4.1	22.0 ± 4.6^a^	24.8 ± 4.0^a,b,c,d^	24.3 ± 4.0^a^	20.6 ± 4.2^e^	23.2 ± 4.6^a^
50°	19.8 ± 3.5	20.9 ± 4.2	21.5 ± 4.6	22.5 ± 5.3^a^	26.0 ± 4.5^a,b,c,d^	26.0 ± 4.5^a^	21.4 ± 4.6^e^	24.3 ± 5.0^a^
60°	19.2 ± 3.4	20.6 ± 4.4	21.2 ± 4.9	22.4 ± 5.7	26.3 ± 4.9^a,b,c,d^	26.5 ± 4.9^a^	21.3 ± 4.7^e^	24.4 ± 5.1^a^
70°	18.7 ± 3.2	20.1 ± 4.3	20.7 ± 4.8	22.1 ± 5.8	26.0 ± 5.1^a,b,c,d^	26.4 ± 5.0^a^	20.6 ± 4.6^e^	23.9 ± 5.1^a^
80°	18.2 ± 3.1	19.7 ± 4.2	20.3 ± 4.7	21.8 ± 5.7^a,b^	25.4 ± 5.1^a,b,c,d^	25.9 ± 5.0^a^	19.8 ± 4.6^e^	23.2 ± 5.0^a^
90°	18.0 ± 2.9	19.4 ± 3.9	20.1 ± 4.4	21.6 ± 5.4^a,b,c^	24.8 ± 4.9^a,b,c,d^	25.3 ± 4.7^a^	19.0 ± 4.8^e^	22.5 ± 4.9^a^
100°	17.9 ± 2.6	19.0 ± 3.5	19.8 ± 4.2	21.6 ± 5.2^a,b,c^	24.5 ± 4.8^a,b,c,d^	24.9 ± 4.7^a^	18.4 ± 5.2^e^	21.7 ± 5.1^a^

^a^Significant difference from intact state (*p* < 0.05)

^b^Significant difference from ACL cut state (*p* < 0.05)

^c^Significant difference from ALL + ACL cut state (*p* < 0.05)

^d^Significant difference from ITB graft harvest + ALL + ACL cut state (*p* < 0.05)

^e^Significant difference from ACL graft state (*p* < 0.05)

Further sectioning of the ALL did not increase tibial internal rotation laxity significantly compared to either the intact knee or the ACL-deficient knee at any flexion angle, with mean changes < 1°.

Harvesting the midportion ITB strip increased tibial internal rotation laxity significantly when compared to the ACL plus ALL-deficient state at 90° and 100° flexion (mean changes < 2°), and the knee was now significantly more lax for internal rotation than when intact across the range 20°–100° flexion.

Additional sectioning of the deep ITB significantly increased internal rotation laxity at 20°–100° flexion compared to the ACL and ALL-deficient knee (*p* ≤ 0.012), with a maximum mean change of 5° at 70° knee flexion.

Thus, after the cutting stages, the knees with combined soft tissue damage (ITB graft harvested + deep ITB + ALL + ACL cut) were significantly more lax in tibial internal rotation than the intact knee, across 10°–100°, *p* ≤ 0.017, with mean laxity increases of 4°–7°.

There was no change in tibial external rotation laxity after cutting the ACL and anterolateral structures.

### The effects of combined anterior translation plus internal rotation loading on tibiofemoral laxity

When applying a combined anterior drawer and internal rotation torque, cutting the ACL significantly increased anterior translation laxity compared to the intact knee from 0° to 60° flexion, but did not increase internal rotation.

Cutting the ALL did not change either the anterior translation or internal rotation at any angle of knee flexion (n.s.) compared to the ACL-deficient knee. Sectioning the deep ITB significantly increased both anterior translation laxity and internal rotation at 20°–100° and 30°–100°, respectively (*p* < 0.05), compared to the ACL and ALL-deficient knee.

Overall, the changes of both tibial anterior translation and internal rotation caused by combined anterior translation plus internal rotation loading were very similar to the patterns of laxity measured under isolated anterior translation or internal rotation loads, but with the translations reduced by approximately 3 mm and the internal rotations increased by approximately 3° across the range of knee flexion examined.

### The ability of isolated ACL and combined ACL plus mLET procedures to restore intact knee laxity

**Fig. 6:**
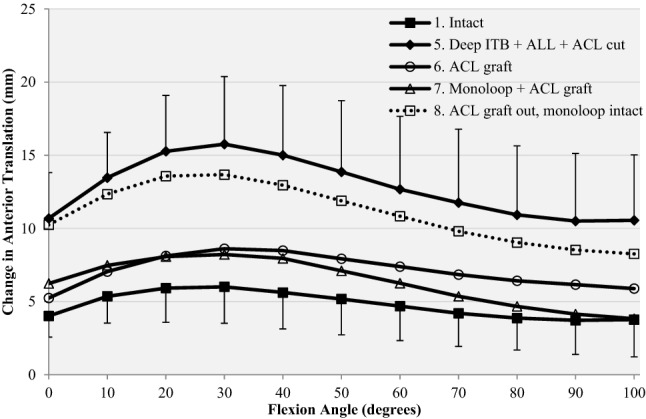
Response to a 90-N anterior drawer force for (1) intact knee, (5) release of deep ITB, section of ACL, ALL and ITB graft harvested (Deep ITB + ACL + ALL cut), (6) ACL reconstruction alone (ACL graft), (7) ACL reconstruction combined with mLET (mLET + ACL graft), (8) mLET alone after removal of the ACL graft (ACL graft out, mLET intact)

After isolated ACL reconstruction, anterior translation laxity did not differ significantly from the intact knee at any angle of knee flexion from 0° to 100° (n.s.), with residual changes from 1 to 3 mm (Fig. 6, Table 1).

Addition of the mLET to the ACL reconstruction maintained anterior translation laxity that did not differ significantly (n.s.) from the intact values at all angles of knee flexion examined.

**Fig. 7:**
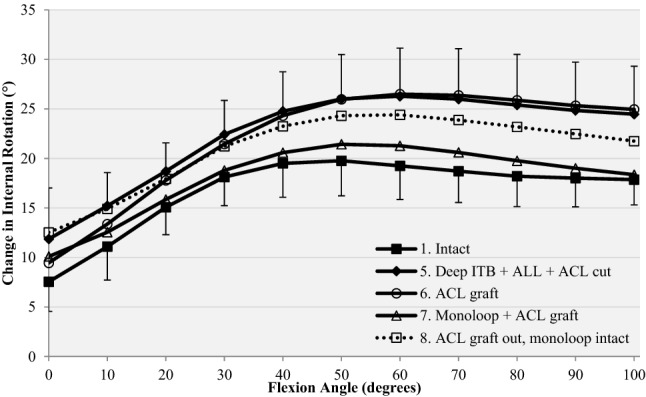
Response to a 5-Nm internal rotation torque for (1) intact, (5) release of deep ITB, section of ACL, ALL and ITB graft harvested (Deep ITB + ALL + ACL cut), (6) ACL reconstruction alone (ACL graft), (7) ACL reconstruction combined with mLET (mLET + ACL graft), (8) isolated mLET after removal of the ACL graft (ACL graft out, mLET intact)

After the isolated ACL reconstruction, internal rotation remained significantly greater than intact knee values at 30°–100° knee flexion (*p* ≤ 0.008, Fig. 7, Table 2).

With the combined ACL reconstruction plus mLET, there were no significant differences in internal rotation compared to the intact knee. Thus, there was a significant decrease in internal rotation laxity after mLET compared to isolated ACL reconstruction at 20°–100° knee flexion (*p* < 0.01, Table 2), and the combined procedure had not overconstrained the internal rotation compared to the intact knee at any angle of knee flexion (Fig. 7, Table 2).

There were no changes in posterior translation or external rotation laxity in the reconstructed states.

With combined anterior drawer and internal rotation torque, after isolated ACL reconstruction there was significantly increased laxity compared to the intact knee in anterior translation at 30°–100° flexion (*p* ≤ 0.035) and internal rotation at 20°–100° flexion (*p* ≤ 0.042).

With combined anterior drawer and internal torque, after additional mLET no significant differences remained in anterior drawer or internal rotation compared to the intact knee.

There was significant decrease in anterior translation laxity after adding the mLET compared to the isolated ACL reconstruction at 20°–80° flexion (*p* ≤ 0.027), and internal rotation at 20°–100° flexion (*p* ≤ 0.016).

### The ability of an isolated mLET to restore intact knee laxity

Without the ACL graft, the knee with an isolated mLET remained significantly more lax in anterior translation than the native knee throughout 0°–100° knee flexion (*p* ≤ 0.009) and in internal rotation from 30° to 100° (*p* ≤ 0.020). These findings were also found under combined anterior drawer and internal torque loading: anterior translation *p* ≤ 0.037, internal rotation *p* < 0.044, except at 20°, n.s. (Figs. 6, 7).

## Discussion

An important finding of this study was that an ACL reconstruction alone was insufficient to restore native knee laxity in a knee with combined ACL deficiency and an injury to the anterolateral structures including the ALL and the deep and superficial fibres of the ITB. This biomechanical study showed that an ACL reconstruction in combination with a monoloop LET restored both tibial anterior translation and tibial internal rotational laxity to the native values in a knee with combined ACL plus anterolateral soft tissue deficiency at time zero. These findings therefore confirmed the initial hypotheses.

### Residual laxity/ALL vs ITB

Residual laxity persisted after isolated ACL reconstruction. Residual laxity after anatomical ACL reconstruction in an ALL, deep and superficial ITB-deficient knee has also been described by Geeslin and Inderhaug [12, 17]. Kittl et al. [21] found that sectioning the ACL plus anterolateral structures caused significant increases of translational and rotational laxity. Similarly, Inderhaug et al. [17] found that sectioning the deep ITB and ALL in an ACL-intact knee caused significant increases in both rotational and translational laxity. It could be speculated that a greater tension in the ACL graft would have reduced this residual laxity at time zero.

A release of the deep ITB fibres significantly increased anterior translation and internal rotational laxity in the knee with ACL and ALL deficiency, whereas cutting the ALL did not, as had been hypothesised. This was also reported by Geeslin et al. [11]. However, Geeslin et al. and Rasmussen et al. [32] found a significant increase of internal rotation with pivot-shift testing of ALL-deficient knees.

While recent literature has often focussed on the ALL as the anterolateral structure to reconstruct when controlling rotational laxity, the focus of attention should concentrate on the deep fibres of the ITB, which are a more substantial structure and have been known for a long time to be important in controlling rotational stability and is seen to be damaged in ACL-deficient knees [39, 42]. The ITB makes a larger contribution to resisting tibial internal rotation than the ALL [11, 21]. An LET graft is aligned more efficiently than an ALL graft to resist anterior movement of the lateral aspect of the tibia, because of its more anterior attachment [2]. Although the ITB is important in controlling rotational laxity, harvesting a midportion ITB strip in the present study had only a minimal effect on rotational laxity, so it appears to be a suitable graft for a lateral tenodesis.

### Combination of ACL reconstruction with ALL or LET

The monoloop LET combined with an ACL reconstruction restored both tibial anterior translation and tibial internal rotational laxity to the native values in a knee with combined ACL plus anterolateral soft tissue deficiency. Geeslin et al. [12] found that combining an ACL reconstruction with an ALL reconstruction or LET could reduce the residual anterior translation after an ACL reconstruction in an ALL and deep ITB deficient knee, and overconstrain internal rotation. This overconstraint was not found with the mLET technique. Inderhaug et al. [17] combined an ACL reconstruction with Lemaire, Macintosh and ALL techniques in ACL, ALL and deep ITB-deficient knees. The tenodeses tensioned at 20 N restored both anterior translational and internal rotational laxity to intact values.[17].

The original MacIntosh [1] and Lemaire [28] procedures both included femoral bone tunnels proximal to the LCL and secured the graft at slightly different points to that used in the present study. However, probably more important is that all three passed the graft deep to the LCL, and so the path was controlled by the attachment of the LCL acting as a pulley near knee extension, leading to a similar function. That path leads to near isometric LET behaviour, with a slight tightening as the knee extends [20]. Reviews of clinical outcomes have shown good results of combined ACL plus lateral procedures [6, 25, 34]. Addition of an extra-articular procedure to an ACL reconstruction significantly reduced the prevalence of residual pivot shift, allowed an earlier return to sports and a better subjective outcome [40, 43]. Thus, the literature supports the addition of a mLET lateral procedure to the intra-articular ACL reconstruction.

### Monoloop alone insufficient

The mLET alone—without an ACL reconstruction—was unable to stabilise the knee sufficiently when tensioned at 20 N. Slette et al. [36] reported that isolated LET reduced anterior translation laxity, but not back to native levels, and at the expense of reduced tibial internal rotation laxity. Tensioning the LET more to control tibiofemoral laxity risks overconstraining the knee, alternating articular contact in the lateral compartment and elevating the contact stresses [16]. Inderhaug et al. found that, when combining an ACL reconstruction with a lateral tenodesis tensioned at 20 N, native knee kinematics were restored, while with 40 N of tension on the tenodesis the laxity was overconstrained in deep flexion [17].

A limitation of the present study was that the cadaveric study was at time zero and therefore did not take into account any healing or stretching out of the damaged structures. The knee specimens were older than in the clinical population, but their ages were in line with those used in other similar studies. To provide a reproducible injury to the anterolateral structures, the protocol involved extensive resection of the fibres with a scalpel. Therefore, the injury state of the knees might be considered a ‘worst-case’ scenario in clinical terms.

## Conclusion

This study found that cutting the deep fibres of the ITB increased tibial internal rotation laxity across the range of knee flexion, while cutting the ALL alone did not. In case of an ACL deficiency combined with increased rotational laxity caused by cutting both the ALL and deep fibres of the ITB, an ACL reconstruction alone was insufficient to restore normal knee laxity. However, adding a ‘monoloop’ lateral extra-articular tenodesis procedure restored the normal knee laxity.

## References

[1] Amirault JD, Cameron JC, MacIntosh DL, Marks P (1988). Chronic anterior cruciate ligament deficiency. Long-term results of MacIntosh's lateral substitution reconstruction. J Bone Jt Surg Br.

[2] Amis AA (2017). Anterolateral knee biomechanics. Knee Surg Sports Traumatol Arthrosc.

[3] Amis AA, Scammell BE (1993). Biomechanics of intra-articular and extra-articular reconstruction of the anterior cruciate ligament. J Bone Jt Surg Br.

[4] Bull AM, Earnshaw PH, Smith A, Katchburian MV, Hassan AN, Amis AA (2002). Intraoperative measurement of knee kinematics in reconstruction of the anterior cruciate ligament. J Bone Jt Surg Br.

[5] Claes S, Vereecke E, Maes M, Victor J, Verdonk P, Bellemans J (2013). Anatomy of the anterolateral ligament of the knee. J Anat.

[6] Colombet PD (2011). Navigated intra-articular ACL reconstruction with additional extra-articular tenodesis using the same hamstring graft. Knee Surg Sports Traumatol Arthrosc.

[7] Cuomo P, Rama KR, Bull AM, Amis AA (2007). The effects of different tensioning strategies on knee laxity and graft tension after double-bundle anterior cruciate ligament reconstruction. Am J Sports Med.

[8] Dodds AL, Gupte CM, Neyret P, Williams AM, Amis AA (2011). Extra-articular techniques in anterior cruciate ligament reconstruction: a literature review. J Bone Jt Surg Br.

[9] Ellison AE (1979). Distal iliotibial-band transfer for anterolateral rotatory instability of the knee. J Bone Jt Surg Am.

[10] Ferretti A, Monaco E, Vadala A (2014). Rotatory instability of the knee after ACL tear and reconstruction. J Orthop Traumatol.

[11] Geeslin AG, Chahla J, Moatshe G, Muckenhirn KJ, Kruckeberg BM, Brady AW, Coggins A, Dornan GJ, Getgood AM, Godin JA, LaPrade RF (2018). Anterolateral knee extra-articular stabilizers: a robotic sectioning study of the anterolateral ligament and distal iliotibial band Kaplan fibers. Am J Sports Med.

[12] Geeslin AG, Moatshe G, Chahla J, Kruckeberg BM, Muckenhirn KJ, Dornan GJ, Coggins A, Brady AW, Getgood AM, Godin JA, LaPrade RF (2018). Anterolateral knee extra-articular stabilizers: a robotic study comparing anterolateral ligament reconstruction and modified Lemaire lateral extra-articular tenodesis. Am J Sports Med.

[13] Godin JA, Chahla J, Moatshe G, Kruckeberg BM, Muckenhirn KJ, Vap AR, Geeslin AG, LaPrade RF (2017). A comprehensive reanalysis of the distal iliotibial band: quantitative anatomy, radiographic markers, and biomechanical properties. Am J Sports Med.

[14] Goldman AB, Pavlov H, Rubenstein D (1988). The Segond fracture of the proximal tibia: a small avulsion that reflects major ligamentous damage. Am J Roentgenol.

[15] Harms SP, Noyes FR, Grood ES, Jetter AW, Huser LE, Levy MS, Gardner EJ (2015). Anatomic single-graft anterior cruciate ligament reconstruction restores rotational stability: a robotic study in cadaveric knees. Arthroscopy.

[16] Inderhaug E, Stephen JM, El-Daou H, Williams A, Amis AA (2017). The effects of anterolateral tenodesis on tibiofemoral contact pressures and kinematics. Am J Sports Med.

[17] Inderhaug E, Stephen JM, Williams A, Amis AA (2017). Biomechanical comparison of anterolateral procedures combined with anterior cruciate ligament reconstruction. Am J Sports Med.

[18] Kennedy MI, Claes S, Fuso FA, Williams BT, Goldsmith MT, Turnbull TL, Wijdicks CA, LaPrade RF (2015). The anterolateral ligament: an anatomic, radiographic, and biomechanical analysis. Am J Sports Med.

[19] Khadem R, Yeh CC, Sadeghi-Tehrani M, Bax MR, Johnson JA, Welch JN, Wilkinson EP, Shahidi R (2000). Comparative tracking error analysis of five different optical tracking systems. Comput Aided Surg.

[20] Kittl C, Halewood C, Stephen J, Gupte CM, Weiler A, Williams A, Amis AA (2015). Length change patterns of the lateral extra-articular structures of the knee and related reconstructions. Am J Sports Med.

[21] Kittl C, El-Daou H, Athwal KK, Gupte CM, Weiler A, Williams A, Amis AA (2016). The role of the anterolateral structures and the ACL in controlling laxity of the intact and ACL-deficient knee. Am J Sports Med.

[22] Kondo E, Merican AM, Yasuda K, Amis AA (2014). Biomechanical analysis of knee laxity with isolated anteromedial or posterolateral bundle-deficient anterior cruciate ligament. Arthroscopy.

[23] Lemaire M (1967). Rupture anciennes du ligament croisé antérieur. Frequence-clinique traitement. J Bone Jt Surg Br.

[24] Mansour R, Yoong P, McKean D, Teh JL (2014). The iliotibial band in acute knee trauma: patterns of injury on MR imaging. Skeletal Radiol.

[25] Marcacci M, Zaffagnini S, Iacono F, Vascellari A, Loreti I, Kon E, Presti ML (2003). Intra- and extra-articular anterior cruciate ligament reconstruction utilizing autogeneous semitendinosus and gracilis tendons: 5-year clinical results. Knee Surg Sports Traumatol Arthrosc.

[26] Matsumoto H (1990). Mechanism of the pivot shift. J Bone Jt Surg Br.

[27] Monaco E, Fabbri M, Mazza D, Daggett M, Redler A, Lanzetti RM, De Carli A, Ferretti A (2018). The effect of sequential tearing of the anterior cruciate and anterolateral ligament on anterior translation and the pivot-shift phenomenon: a cadaveric study using navigation. Arthroscopy.

[28] Neyret P, Palomo JR, Donell ST, Dejour H (1994). Extra-articular tenodesis for anterior cruciate ligament rupture in amateur skiers. Br J Sports Med.

[29] Nitri M, Rasmussen MT, Williams BT, Moulton SG, Cruz RS, Dornan GJ, Goldsmith MT, LaPrade RF (2016). An in vitro robotic assessment of the anterolateral ligament, part 2: anterolateral ligament reconstruction combined with anterior cruciate ligament reconstruction. Am J Sports Med.

[30] Noyes FR, Barber SD (1991). The effect of an extra-articular procedure on allograft reconstructions for chronic ruptures of the anterior cruciate ligament. J Bone Jt Surg Am.

[31] Rahnemai-Azar AA, Naendrup JH, Soni A, Olsen A, Zlotnicki J, Musahl V (2016). Knee instability scores for ACL reconstruction. Curr Rev Musculoskelet Med.

[32] Rasmussen MT, Nitri M, Williams BT, Moulton SG, Cruz RS, Dornan GJ, Goldsmith MT, LaPrade RF (2016). An in vitro robotic assessment of the anterolateral ligament, part 1: secondary role of the anterolateral ligament in the setting of an anterior cruciate ligament injury. Am J Sports Med.

[33] Reid JS, Hanks GA, Kalenak A, Kottmeier S, Aronoff V (1992). The Ellison iliotibial-band transfer for a torn anterior cruciate ligament of the knee. Long-term follow-up. J Bone Jt Surg Am.

[34] Rezende FC, de Moraes VY, Martimbianco AL, Luzo MV, da Silveira-Franciozi CE, Belloti JC (2015). Does combined intra- and extraarticular ACL reconstruction improve function and stability? A meta-analysis. Clin Orthop Relat Res.

[35] Samuelson M, Draganich LF, Zhou X, Krumins P, Reider B (1996). The effects of knee reconstruction on combined anterior cruciate ligament and anterolateral capsular deficiencies. Am J Sports Med.

[36] Slette EL, Mikula JD, Schon JM, Marchetti DC, Kheir MM, Turnbull TL, LaPrade RF (2016). Biomechanical results of lateral extra-articular tenodesis procedures of the knee: a systematic review. Arthroscopy.

[37] Sonnery-Cottet B, Lutz C, Daggett M, Dalmay F, Freychet B, Niglis L, Imbert P (2016). The involvement of the anterolateral ligament in rotational control of the knee. Am J Sports Med.

[38] Suero EM, Njoku IU, Voigt MR, Lin J, Koenig D, Pearle AD (2013). The role of the iliotibial band during the pivot shift test. Knee Surg Sports Traumatol Arthrosc.

[39] Terry GC, Norwood LA, Hughston JC, Caldwell KM (1993). How iliotibial tract injuries of the knee combine with acute anterior cruciate ligament tears to influence abnormal anterior tibial displacement. Am J Sports Med.

[40] Vadalà AP, Iorio R, De Carli A, Bonifazi A, Iorio C, Gatti A, Rossi C, Ferretti A (2013). An extra-articular procedure improves the clinical outcome in anterior cruciate ligament reconstruction with hamstrings in female athletes. Int Orthop.

[41] Woods GW, Stanley RF, Tullos HS (1979). Lateral capsular sign: X-ray clue to a significant knee instability. Am J Sports Med.

[42] Yamamoto Y, Hsu WH, Fisk JA, Van Scyoc AH, Miura K, Woo SL (2006). Effect of the iliotibial band on knee biomechanics during a simulated pivot shift test. J Orthop Res.

[43] Zaffagnini S, Marcacci M, Lo Presti M, Giordano G, Iacono F, Neri MP (2006). Prospective and randomized evaluation of ACL reconstruction with three techniques: a clinical and radiographic evaluation at 5 years follow-up. Knee Surg Sports Traumatol Arthrosc.

